# Tryptophan Metabolism in Inflammaging: From Biomarker to Therapeutic Target

**DOI:** 10.3389/fimmu.2019.02565

**Published:** 2019-10-30

**Authors:** Freek J. H. Sorgdrager, Petrus J. W. Naudé, Ido P. Kema, Ellen A. Nollen, Peter P. De Deyn

**Affiliations:** ^1^European Research Institute for the Biology of Ageing, University Medical Center Groningen, University of Groningen, Groningen, Netherlands; ^2^Laboratory of Neurochemistry and Behavior, Department of Biomedical Sciences, Institute Born-Bunge, University of Antwerp, Antwerp, Belgium; ^3^Department of Neurology and Alzheimer Center, University Medical Center Groningen, University of Groningen, Groningen, Netherlands; ^4^Department of Laboratory Medicine, University Medical Center Groningen, University of Groningen, Groningen, Netherlands; ^5^Department of Molecular Neurobiology, Groningen Institute for Evolutionary Life Sciences (GELIFES), University of Groningen, Groningen, Netherlands; ^6^Department of Neurology, Memory Clinic of Hospital Network Antwerp (ZNA) Middelheim and Hoge Beuken, Antwerp, Belgium

**Keywords:** tryptophan, aging, inflammation, kynurenine, inflammaging, tryptophan 2,3-dioxygenase (TDO), indoleamine 2,3 dioxygenases (IDO)

## Abstract

Inflammation aims to restore tissue homeostasis after injury or infection. Age-related decline of tissue homeostasis causes a physiological low-grade chronic inflammatory phenotype known as inflammaging that is involved in many age-related diseases. Activation of tryptophan (Trp) metabolism along the kynurenine (Kyn) pathway prevents hyperinflammation and induces long-term immune tolerance. Systemic Trp and Kyn levels change upon aging and in age-related diseases. Moreover, modulation of Trp metabolism can either aggravate or prevent inflammaging-related diseases. In this review, we discuss how age-related Kyn/Trp activation is necessary to control inflammaging and alters the functioning of other metabolic faiths of Trp including Kyn metabolites, microbiota-derived indoles and nicotinamide adenine dinucleotide (NAD^+^). We explore the potential of the Kyn/Trp ratio as a biomarker of inflammaging and discuss how intervening in Trp metabolism might extend health- and lifespan.

## Inflammaging: Chronic Inflammation That Drives the Aging Process

Inflammation is initiated by the innate immune system in response to mechanical, infectious, or metabolic tissue stress and aims to restore homeostasis by eliminating damaged cells (1). Aging is characterized by progressive decline of tissue homeostasis resulting from damaged cellular components and aberrant functioning of damage-response mechanisms (2).

Age-related changes of the innate immune system are common and include shifts in the composition of immune cell populations, altered secretory phenotypes and impaired signaling transduction (3). These changes are paralleled by the development of a chronic inflammatory state referred to as *inflammaging*. This is characterized by an imbalance between pro- and anti-inflammatory responses and fluctuations of inflammatory cytokines, such as interleukin-6 (IL-6), high-sensitive C reactive protein (hsCRP), IL-10 and tissue growth factor beta (TGF-β) (4, 5). The rate of inflammaging, quantified by measuring these markers, is strongly associated with age-related disability, disease and mortality (6). It is theorized that inflammaging is driven by endogenous ligands released upon age-related tissue damage and can be aggravated by food excess and attenuated by caloric restriction, suggesting relevant cross-talk between metabolic and immune functioning (7).

Understanding how inflammaging is controlled could aid in the development of diagnostic and therapeutic tools for many age-related diseases associated with inflammation such as cancer, atherosclerosis, diabetes mellitus, and Alzheimer's disease. Tryptophan (Trp) metabolism is associated with aging and produces metabolites that control inflammation, regulate energy homeostasis and modulate behavior (8). We discuss how activation of Trp metabolism could be involved in the control of inflammaging and how this can alter the Trp metabolite milieu. We hypothesize on how this could impact health- and lifespan and how interfering with Trp metabolism could be used in the treatment of neurodegenerative diseases.

## Activation of Tryptophan Metabolism Regulates Inflammation

### Inflammation Activates Tryptophan Metabolism

The essential amino acid Trp fuels the synthesis of kynurenine (Kyn), serotonin (5-HT) and indoles (9, 10). The Kyn pathway of Trp is the most active pathway of Trp metabolism and produces metabolites including kynurenic acid and nicotinamide adenine dinucleotide (NAD^+^). The Kyn pathway is initiated by the enzymes tryptophan 2,3-dioxygenase (TDO) and indoleamine 2,3-dioxygenase (IDO and IDO2). In this review, we focus on the role of IDO1, which we refer to as IDO. Expression of TDO and IDO (and other enzymes in the Kyn pathway) is species-, cell type-, and context-specific (11–13).

While IDO plays a minor role in Trp metabolism under normal circumstances, IDO-dependent Trp metabolism is strongly activated in response to interferons and other cytokines that are released upon inflammation (14). Interferon gamma (IFN-γ) is considered the most potent IDO-activating cytokine and induces expression in a variety of cell types after it binds to the IDO promotor-region. The effect of IFN-γ on IDO activation is best-characterized in macrophages and dendritic cells (DCs) but is also evident in connective (e.g., fibroblast) and epithelial tissue (e.g., pulmonary, renal, gastro-intestinal, and vascular) (15–19).

Other inflammatory signals that activate IDO include lipid mediators such as prostaglandin E2 (PGE2) and pathogen particles such as lipopolysaccharides (LPS) (20). In addition, while the regulation of IDO is often transcriptional, specific mediators of inflammation induce post-transcriptional and post-translational modifications that either promote ubiquitination and proteasomal degradation of IDO or sustain its activity through phosphorylation (21, 22).

Inflammation-related IDO activity is often measured by the Kyn/Trp ratio in blood in diseases characterized by excessive or chronic inflammation including infections, auto-immune disorders, cardiovascular disease, and cancer (23).

### Activation of Tryptophan Metabolism Has Anti-inflammatory and Immunosuppressive Effects

**Figure 1:**
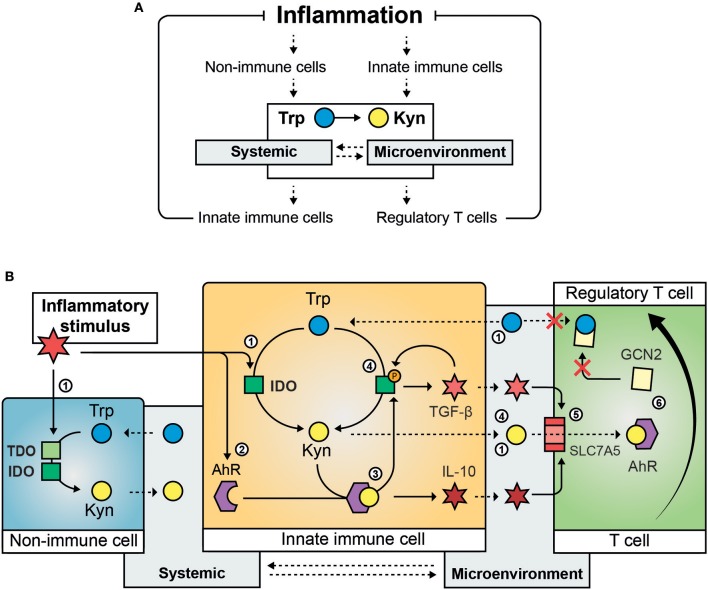
Mechanisms involved the regulation of inflammation by Trp metabolism. Inflammation activates Trp metabolism and causes systemic and intra- and extra-cellular changes in the Kyn/Trp ratio that suppress the inflammatory response **(A)**. The molecular steps involved in the immunomodulatory effect of activation of Trp metabolism **(B)**: An inflammatory stimulus activates IDO (and in specific instances TDO) in immune and non-immune cells causing reduced Trp systemic and local Trp levels and increased intra- and extracellular Kyn content (1); inflammation induces increased expression of *AhR* (2) that is activated by its ligand Kyn and results in the secretion of anti-inflammatory cytokines such as IL-10 (3); AhR ligand-activation causes phosphorylation of IDO and results in sustained IDO activity and the secretion of TGF-β, which is involved in a feedback loop by inducing IDO phosphorylation (4); inflammatory cytokines such as TGF-β and IL-10 induce the amino acid transporter SLC7A5 on the plasma membrane of naïve T-cells causing transport of Kyn into the T cell (5); activation of GCN2 by Trp depletion and AhR ligand-activation by Kyn cause the differentiation of naïve T cells toward regulatory T cells (6). Solid arrows indicate regulatory (transcriptional or translational) and enzymatic effects, dashed arrows indicate active or passive cross-cellular and cross-compartmental transport of Trp and Kyn. Trp, Tryptophan; Kyn, Kynurenine, IDO, indoleamine 2,3-dioxygenase; TDO, tryptophan 2,3-dioxygenase; AhR, aryl hydrocarbon receptor; TGF-β, tissue growth factor beta; IL-10, interleukin 10; SLC7A5, solute carrier family 7 member 5; GCN2, general control non-derepressable 2 stress kinase.

Trp metabolism controls hyperinflammation and induces long term immune tolerance. These effects pivot on the ability of IDO to alter the local and systemic Kyn/Trp balance (Figure 1A). This balance directly affects metabolic and immune signaling pathways that drive an anti-inflammatory response in IDO-competent cells (e.g., antigen-presenting cells and epithelial cells). In addition, it changes the function of neighboring cells (e.g., T cells) by creating a local (and sometimes systemic) environment high in Kyn and low in Trp. Several molecular pathways mediate immune and non-immune responses to changes in intracellular Trp and Kyn levels (Figure 1B).

#### Trp Depletion in the Metabolic Regulation of Inflammation and Tolerance

Trp levels influence nutrient sensing systems such as the general control non-derepressable 2 (GCN2) stress kinase and mechanistic target of rapamycin complex 1 (mTORC1). The kinase GCN2 is activated during amino acid depletion (or imbalance) and causes phosphorylation of eukaryotic initiation factor (eIF)2α that has cell-type specific effects on translation. mTORC1 is active during amino acid sufficiency and governs anabolic metabolism and energy expenditure. GCN2 and mTORC1 are implicated in the metabolic control of inflammation by immune and non-immune cells (24).

Trp depletion activates GCN2 in IDO-expressing dendritic cells and macrophages causing them to produce anti-inflammatory cytokines including interleukin-10 (IL-10) and TGF-β instead of immunogenic cytokines (25, 26). Additionally, Trp depletion can alter the secretory phenotype of neighboring IDO-incompetent dendritic cells, cause GCN2-dependent differentiation and recruitment of regulatory T cells (T_reg_) (27, 28) and prevent T cell activation and proliferation (25). These concepts seem to be involved in providing tolerance to apoptotic cells in the spleen (26, 29). However, the role of IDO/GCN2-signaling is not limited to immune cells. In an antibody-induced model for glomerulonephritis in mice, which is lethal in mice lacking *IDO* expression, IDO/GCN2 signaling limited inflammatory tissue damage by inducing autophagy in renal epithelial cells (15). Taken together, these studies indicate that IDO can prevent inflammation and promote tolerance in a context-specific manner by regulating GCN2 activity in immune and non-immune cells.

mTORC1 is a central regulator of cellular function. Cells of the innate immune system largely depend on mTORC1 to enable the metabolic transition that is required for their activation (30). mTORC1 orchestrates the cellular immune behavior in response to extracellular and intracellular factors such as inflammatory stimuli, glucose availability and amino acid sufficiency. *In vitro* studies showed that IFN-γ inhibited mTORC1 by depleting cellular Trp levels in IDO-expressing cells (31) causing suppression of mTORC1 co-localization to the lysosome and altering the metabolic functioning of human primary macrophages (32). The relevance of IDO/mTORC1 signaling in controlling inflammation *in vivo* is yet to be established.

Future studies are needed to determine how the cellular Trp content is regulated in response to exogenous and endogenous inflammatory stimuli and how Trp levels affect GCN2 and mTORC1 signaling to determine the metabolic control of inflammation *in vivo*.

#### Kyn Activates the Aryl Hydrocarbon Receptor

Activated Trp metabolism results in increased Kyn production. The role of Kyn in the regulation of inflammation is largely mediated through its function as a ligand of the aryl hydrocarbon receptor (AhR), a transcription factor that controls local and systemic immune responses. Recent studies are suggesting that Kyn/AhR signaling is involved in the generation of T_reg_ cells and the modulation of the immune phenotype of DCs.

T_reg_ cells are derived from naïve T cells and are involved in maintenance of immunological tolerance but also aid macrophages during the resolution of inflammation by stimulating them to secrete anti-inflammatory cytokines (33) and aging is associated with increased T_reg_ populations in immune and non-immune tissue (34). Kyn supplementation can activate AhR in naïve T cells in the presence of specific inflammatory cytokines and directly drive T_reg_ differentiation (35). Although Kyn passes relatively easily across the cell membrane of most cell types, recent data suggest that Kyn-dependent AhR activation in T cells requires Kyn transport across the amino acid transporter SLC7A5, which is expressed upon T cell activation (36). DCs play an essential role in creating the microenvironment that is required for T_reg_ differentiation. To do so, DCs take on a specific secretory phenotype that is also driven by Kyn-dependent AhR activation by Nguyen et al. (37). Interestingly, AhR activation can also induce the expression of *IDO*, suggesting a Kyn/AhR/IDO feedback loop that is possibly involved in the maintenance of an immunosuppressive phenotype in DCs (38).

IDO function in DCs seems to be sustained by phosphorylation caused either by a chaperone of AhR that is released upon Kyn binding (39) or through autocrine TGF-β and NF-κB dependent signaling (22). In the latter study, IDO seemed to act through a non-catalytic mechanism. In both studies, IDO phosphorylation sustained the immunomodulatory phenotype of DCs necessary for long-term tolerance to inflammatory stimuli. As this type of tolerance could be required to dampen age-related inflammation, it would be of great interest to study IDO phosphorylation in aged immune tissue.

To conclude, IDO/Kyn/AhR signaling can modulate the innate immune system to create an anti-inflammatory microenvironment that is favorable for the generation of T_reg_ cells and critical for the maintenance of long-term immunosuppression.

### Tryptophan Metabolism Controls Inflammation *in vivo*

The important role of Trp metabolism in controlling inflammation is highlighted by studies in IDO deficient mice. These mice show no apparent inflammatory phenotype or auto-immune disorders (within controlled, pathogen-free laboratory facilities). Yet, when confronted with an inflammatory stimulus they develop severe inflammatory diseases. These include pulmonary infections in response to stem cell transplantation (40), antibody-induced renal inflammation (15), auto-immunity in response to chronic exposure to apoptotic cells (29), severe colitis in response to 2,4,6-trinitrobenzene sulfonic acid (17), aggravation of hepatic inflammation in response to a high-fat diet (41) and aggravation of hypercholesterolemia-related atherosclerosis (42). Of note, IDO-deficiency protected from inflammation in a mouse model of chronic gastric inflammation by modulating B cell immunity and suppressing cytotoxicity of natural killer cells (43). The fact that IDO seems to control inflammation in response to so many non-infectious stimuli including metabolic stress, underlines its function as a general regulator of inflammation and suggests that it could be involved in the regulation of inflammaging.

### Other Tryptophan Metabolites Involved in Inflammation

Other Trp metabolites are also involved in the control of inflammation and tissue damage. Examples of this include serotonin, implicated in intestinal inflammation (44); kynurenic acid, which exerts anti-inflammatory changes in adipose tissue (45); 3-hydroxyanthranilic acid and cinnabarinic acid (two other Kyn metabolites) that are, respectively, connected to vascular inflammation (46) and autoimmune encephalomyelitis (47); NAD^+^, which prevents renal kidney injury (48, 49) and regulates macrophage immune responses (50); and indoles, crucially involved in gastro-intestinal and neuronal inflammation (51).

Although a discussion of the specific roles of these metabolites in age-related inflammation is outside the scope of this review, it is important to consider the broad role of Trp metabolism in inflammation.

## Tryptophan Metabolism as a Biomarker and Therapeutic Target in Inflammaging

There is limited evidence of a direct, mechanistic, role of Trp metabolism in inflammaging. Yet, observational studies have indicated that Trp metabolism could be a biomarker for inflammaging. In addition, Trp metabolism could provide therapeutic targets to treat age-related diseases associated with inflammation and possibly even extend lifespan.

### The Kyn/Trp Ratio as a Biomarker for Inflammaging

The Kyn/Trp ratio, measured in blood, is robustly associated with aging in humans (Table S1) (52–60). The fact that this association is already evident in healthy young adults (61) and persists throughout life (56), implies that the age-dependent increase in the Kyn/Trp ratio is not secondary to the onset of disease but rather represents a physiological age-related change. In addition, markers of immune activation are, already in young adults, strongly associated with the Kyn/Trp ratio (62). Taken together, these observational data suggests that the Kyn/Trp ratio could provide a valuable marker for the rate of (physiological) inflammaging.

As inflammaging is involved in the onset of age-related diseases, a marker for inflammaging should also predict the onset of age-related diseases. This is the case for the Kyn/Trp ratio. For example, an increased Kyn/Trp ratio was found to be associated with increased frailty (63), reduced cognitive performance (64), increased risk of cardiovascular disease (65, 66) and mortality (56, 66) in aged individuals. Other Kyn metabolites, including the 3-hydroxyanthranilic acid/anthranilic acid ratio and kynurenic acid, have also been associated with inflammation and poor outcome in the context of (age-related) diseases of the brain (67, 68).

The Kyn/Trp ratio—and potentially other Kyn pathway metabolites—could thus be valuable readouts of the rate of physiological inflammaging in healthy individuals and predict the onset of age-related diseases associated with chronic inflammation. In addition, the Kyn/Trp ratio meets the criteria for a biological age biomarker (as opposed to chronological age) (69). As a single biomarker is seldomly able to predict complex biological processes, the use of the Kyn/Trp ratio in the prediction of inflammaging and biological aging should be validated in concordance with other potential biomarkers of aging preferably in combination with immune markers for sustained inflammation [e.g., GlycA (70)]. These studies should ideally address intraindividual variability of such markers by making use of longitudinal study designs.

### Consequences of Kyn/Trp Shunt in Inflammaging

**Figure 2:**
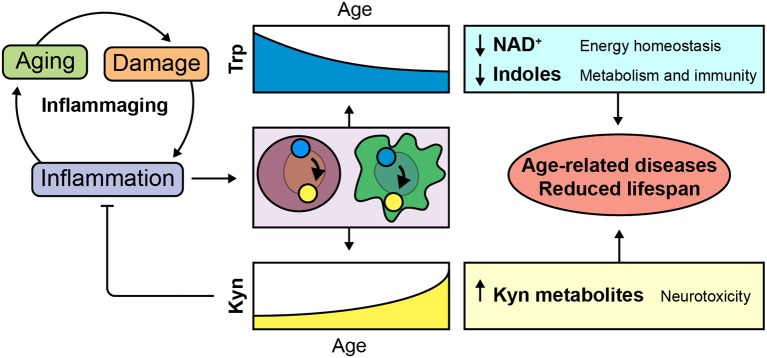
Implications of inflammaging-dependent shunt of Trp metabolism. Age-related decline of tissue homeostasis causes a physiological low-grade chronic inflammatory phenotype known as inflammaging. We hypothesize that Trp is metabolized toward the Kyn pathway in order to control age-related inflammation. Consequent disturbances of Trp and Kyn metabolites could be involved in age-related diseases and reduced lifespan.

An inflammaging-related shunt of Trp metabolism toward extra-hepatic Kyn production could impact the functioning of Trp metabolites in a range of organs during aging (Figure 2).

#### Indoles in Gastro-Intestinal and Metabolic Functioning

The microbiome is increasingly recognized to play an important role in aging and age-related disease (71). Indoles are microbiota-derived Trp metabolites that are implicated in immune regulation and affect gastro-intestinal functioning (51). A recent paper showed that dietary-induced obesity increased intestinal IDO activity shifting Trp metabolism toward the production of Kyn and away from microbiota-derived metabolites (72). Inhibition of IDO in the gut improved insulin sensitivity and resulted in reduced chronic inflammation. In addition, age-related changes to the microbiome were associated with increased expression of enzymes involved in microbial Trp metabolism (73). These data highlights the importance of microbiota-dependent Trp metabolism and suggest that activation of intestinal IDO and age-related changes in microbiome composition can deplete the body of health-promoting indoles while affecting the systemic Kyn/Trp balance. In addition, it provides relevant evidence that links metabolic inflammation (metaflammation) to gastro-intestinal Trp metabolism and metabolic health. In this context, it is interesting to note that Trp metabolites and indoles are emerging as modulators of adipose tissue homeostasis and obesity (45, 74, 75). Age-related gastro-intestinal metaflammation could thus cause metabolic disturbances through altering microbiome and host Trp metabolism.

#### De novo NAD^+^ Synthesis in Age-Related Tissue Decline

The liver metabolizes the majority of Trp in a TDO-dependent manner producing NAD^+^ or acetoacetyl-CoA (9). NAD^+^ is a coenzyme and cosubstrate for several important regulatory proteins involved in cellular metabolism and damage such as sirtuins and Poly(ADP-ribose) polymerases (PARPs). NAD^+^ can be generated *de novo* from Trp or through salvage pathways. While *in vitro* the contribution of *de novo* NAD^+^ synthesis is limited, *in vivo* NAD^+^ is actively synthesized *de novo* from Trp, especially in the liver and the kidney (76).

Declining cellular NAD^+^ content is a cross-species phenotype of aging that is associated with a range of age-related diseases (77). Boosting *de novo* synthesis of NAD^+^ from Trp in the liver—by blocking acetoacetyl-CoA production—improved hepatic function and inflammation in mice on a high fat diet through modulation of mitochondrial function (78). Similarly, increasing *de novo* synthesis of NAD^+^ was protective in mouse models of renal damage (49, 78) and restored age-related functional decline of macrophages (50). These recent studies underline the relevance of *de novo* NAD^+^ synthesis in modulating health and lifespan by regulating mitochondrial function in metabolically active tissue such as immune cells and the liver. Inflammaging could shunt Trp metabolism toward extrahepatic tissue and possibly contribute to age-related hepatic NAD^+^ deficits, providing new evidence for theories that link age-related inflammation and metabolic dysfunction (7).

#### Peripheral Trp Metabolism as a Target for Neurodegenerative Diseases

*TDO2* and *IDO* expression in the brain is low and restricted to specific brain regions. Trp metabolism in the brain is therefore largely dependent on transport of Trp and Kyn across the blood-brain barrier. Modulating peripheral Trp metabolism can thus alter the functioning of Trp and Trp metabolites in the brain (13). In mouse models of Alzheimer's disease and Huntington's disease peripheral inhibition of the Kyn pathway prevented neurodegeneration and memory-deficits (79, 80). Similarly, inhibition of TDO was neuroprotective in fly and worm models of Alzheimer's and Parkinson's disease (81–83). Although the mechanisms that underlie these findings are largely unknown and are difficult to study due to cell type-specific expression of Kyn pathway enzymes in the brain, they could involve a direct effect on protein aggregation, altered immune responses, changed mitochondrial function, or variations in levels of kynurenic acid–a modulator of neurotransmission (13). In addition, the long-term activation of AhR potentially contributes to vascular aging, which is a known risk factor for neurodegenerative diseases (84).

#### Trp in the Regulation of Lifespan

Evidence from studies in *Caenorhabditis elegans* and rodents suggests that targeting Trp metabolism could extend lifespan. For example, we showed that knockdown of *tdo-2* in *C. elegans* increased lifespan with ~15% (83). This effect was dependent on *daf-16*, the *C. elegans* homolog of the forkhead box protein O (FOXO) family of transcription factors. Accordingly, TDO inhibition and Trp feeding extended lifespan in other studies in a daf-16-dependent manner (85, 86).

In rats Trp content in liver, kidney and brains decreases with age while Kyn content in these organs increases (87). A study across 26 mammalian species showed that the Kyn/Trp ratio in the liver of healthy adult animals was associated with species-specific maximum lifespan (88); species that showed a higher Kyn/Trp ratio were shorter lived.

As TDO inhibitors are readily available and TDO knockout mice are viable, these models could be used to study the effects of TDO inhibition on lifespan. However, caution should be warranted as inhibition of Trp metabolism could aggravate immune responses upon inflammatory stimuli (not present in a laboratory context) and may lead to an exacerbated inflammatory environment during inflammaging, which could have dire consequences on health. In addition, failure of recent clinical trials with IDO inhibitors in cancer have underlined that a more thorough understanding of the physiological functions of the Kyn pathway is needed to successfully target the Kyn pathway in disease (10).

## Conclusion

Trp metabolism regulates inflammation, energy homeostasis, and brain functioning. Age-related chronic inflammation—inflammaging—shunts Trp metabolism toward its immunomodulatory catabolite Kyn. Alterations of other Trp metabolites, as a consequence of this adaptive anti-inflammatory mechanism, could drive aging and underlie pathophysiology of age-related diseases. Future studies should address the value of Trp metabolism as a biomarker for (un)healthy aging and as drug target for inflammaging-related disease.

## Author Contributions

FS managed the literature searches and wrote the first draft of the manuscript. PN, IK, EN, and PD critically reviewed the manuscript.

### Conflict of Interest

The authors declare that the research was conducted in the absence of any commercial or financial relationships that could be construed as a potential conflict of interest.
