# Supplementary material for: Tryptophan Metabolism in Inflammaging: From Biomarker to Therapeutic Target
Source: Front Immunol. 2019 Oct 30;10:2565. doi: 10.3389/fimmu.2019.02565 (PMC6833926; doi:10.3389/fimmu.2019.02565)
Supplement: Supplementary file 1 [file Table_1.DOCX]

| **Study** | **Age range** | **N** |  | **Association with aging** |  |
| --- | --- | --- | --- | --- | --- |
|  |  |  | **Trp** | **Kyn** | **Kyn/Trp** |
| (Ramos-Chávez et al., 2018) | 51-97 | 77 | ↓ | na | ↑ |
| (Rist et al., 2017) | 18 - 80 | 301 | ↓ | na | na |
| (Theofylaktopoulou et al., 2013) | 45 - 72 | 7052 | ↓ | ↑ | ↑ |
| (Collino et al., 2013) | 24 - 111 | 254 | ↓ | na | na |
| (Yu et al., 2012) | 32-81 | 2162 | ↓ | na | na |
| (Capuron et al., 2011b) | > 65 | 284 | ↓ | ↑ | ↑ |
| (Niinisalo et al., 2008) | 46 - 76 | 921 | na | na | ↑ |
| (Pertovaara et al., 2006) | 21 - 99 | 593 | = | ↑ | ↑ |
| (Frick et al., 2004) | 34 - 93 | 43 | na | na | ↑ |

**Table S1. Age-related Kyn/Trp changes.** An overview of studies that investigated the effect of age on Trp, Kyn and the Kyn/Trp ratio. The age-range, number of participants and association of Trp, Kyn and Kyn/Trp with aging is provided.

Abbreviations: na, not available
